# A glycemic diet improves the understanding of glycemic control in diabetes patients during their follow-up

**DOI:** 10.2144/fsoa-2022-0058

**Published:** 2023-03-17

**Authors:** Sarvesh Sabarathinam

**Affiliations:** 1 Interdisciplinary Institute of Indian System of Medicine (IIISM), SRM Institute of Science & Technology, Kattankulathur, 603203, Chengalpattu, Tamil Nadu, India

## Overview

Diabetes is a complex metabolic disorder with a high prevalence of macrovascular and microvascular complications. Patients' knowledge and comprehension of the condition are often inadequate, which may cause them to identify complications more slowly than they should. Most diabetic patients are unaware that uncontrolled diabetes may lead to severe complications such as heart attack/foot ulcers and renal problems. Effective communication between doctors and patients on diabetic treatment is critical to glycemic management. It encourages patients to follow-up with their medications regularly and helps glycemic control through dietary modifications. Social and psychological difficulties are disproportionately prevalent among diabetic patients. As a first step in creating programmatic interventions for diabetes, it is essential to recognize and comprehend the challenges physicians face. From the physician's perspective, the Type 2 diabetes epidemic presents many potential patient-related challenges regarding glycemic control. Such challenges include implementing dietary interventions in healthcare settings, limited resources, such as short consultation times, ineffective communication, language barriers, irregular follow-ups and mismatches in patients' or their caretakers' previous medical and medication histories [1].

## Glycemic diet

A controlled glycemic index can be achieved by following a low-fat diet, where fat intake (mainly saturated and cholesterol) is restricted. A low glycemic index diet is where the amount of carbohydrates is adjusted to slow down their absorption in the body. A deficient carbohydrate diet is where protein and fat intake is encouraged. Weight loss is one of the significant risk factors for developing diabetes mellitus and a prominent obstacle in the effective management of diabetes mellitus. Thus, it should be strategically handled. The common practice among obese or diabetic patients to regulate their glycemic level by weight loss is to follow a diet pattern and reduce energy intake. However, this can increase appetite and overeating, resulting in weight gain. A low glycemic index diet causes significant changes in fasting blood glucose levels, HbA1c, total cholesterol and low-density lipoprotein levels.

In a low glycemic index diet, the American diabetes association has recommended that the daily intake of carbohydrates is <130 g. The patients should be restricted from taking food rich in starch and other forms of sugar like refined carbohydrates, grains and certain fruits and vegetables. A low glycemic loaded diet (LGID) encourages patients to consume sources of phytochemicals and phytonutrients rich in amino acids and essential fatty acids. Additionally, patients are asked to take protein and a healthy source of fat as per their preference. A low glycemic index diet also includes eating cruciferous vegetables as they are rich in sulforaphane, sinigrin, glucobrassicin, glucophasatin and glucoiberin. Additionally, indirect antioxidants can combat oxidative stress by acting on the Nrf2 pathway and inhibiting NF-κB in cardiometabolic disorders (dyslipidemia, insulin resistance, impaired glucose intolerance, central adiposity and hypertension) [2].

A reduction in body weight was found to be more effective in people with standard glucose tolerance. In contrast, in people with impaired glucose tolerance and diabetes, there was no improvement in body weight reduction. Existing reports indicate that a low glycemic diet and certain lifestyle modifications can regulate HbA1c, body mass index and lipid profiles in the population above. Previous studies suggest that following a low glycemic index diet can prevent gestational diabetes mellitus (GDM) related complications like macrosomia and significantly reduce fasting and postprandial glucose levels. The highlight is that a low glycemic index diet can reduce the risk of insulin usage [3].

## Role of clinical pharmacist

It has long been understood that coherent communication, team-based treatment, and well-coordinated care strategies are essential to managing chronic diseases. The American Diabetes Association, in their annual Standards of Medical Care in Diabetes, recommended a team-based approach to diabetes care, where healthcare professionals from various backgrounds were led by a physician to improve diabetes care. This diabetes team involved health professionals such as nurses, certified diabetes educators, physician assistants and pharmacists [4,5]. Among the pharmacists, the clinical pharmacist was the most experienced professional in clinical care. Clinical pharmacist services have successfully reduced direct medical costs, improved clinical outcomes, achieved quality measures, and resolved medication-related issues in various chronic disease states, including diabetes and hypertension. This success has been attributed to the widespread recognition of the value of clinical pharmacists. The clinical pharmacist has various roles in the diabetes care team, which include clinical pharmacist ward rounds, patient education, medication adherence monitoring, health barrier assessments, prevention, screening and maintenance.

### Clinical pharmacist ward rounds

The clinical pharmacist visits the diabetes wards before the physicians' control round to review the patient's glycaemic levels from the logbook. They will discuss with the patient to evaluate their practice on therapies, glucose monitoring, diet, and exercise. The intervention for each patient will be noted and intimated to the diabetes care team [6–8].

### Patient education

Patient education is a critical part of diabetes care. The clinical pharmacist will provide education to the diabetes patient after evaluating their clinical and medication charts and also identifying the core areas of patient difficulty [9].

### Medication adherence monitoring & counseling

A clinical pharmacist will analyze the medication chart of each patient and the patient's adherence to their medication, glycemic control, lifestyle modifications, and exercise. Based on the intervention, necessary counseling will be given to patients based on their adherence scale [10].

### Health barriers assessment

A thorough understanding of health barriers is necessary to provide adequate diabetes care. The clinical pharmacist will spend valuable time with the patient to discuss and understand the health barriers, including psychosocial state, financial status, educational status and family status. Identifying the health barriers of each patient will help the diabetes care team to design a personalized care plan [11].

### Prevention, screening & maintenance

A clinical pharmacist will actively participate in prevention and screening activities in inpatient and outpatient environments. Diabetic patients will receive education on prevention and screening and on lifestyle, diet, and addiction. Effective communication between doctors and patients on diabetic treatment is critical to glycemic management. It makes patients regularly follow-up on their medications and helps in glycemic control via diet modifications. A controlled glycemic index can be achieved by following a low glycemic index diet (>130 g/d) where the amount of carbohydrate is altered to slow down the absorption process and a deficient carbohydrate diet. Weight loss is one of the significant risk factors for developing diabetes mellitus, and a prominent obstacle in the effective management of diabetes mellitus should be strategically handled [12].

## Low glycemic foods available in India locally

### Low glycemic index Indian pulses

#### Chana dhal

The glycemic index of chana or Bengal gram, commonly known as white chickpea, is 8. In 100 g, chana dhal contains proteins (14.5 g), calories (269 kcal), fat (4 g), carbs (45 g), vitamins, minerals, and fiber (12–13 g). This can assist in reducing the habit of binge eating by keeping the person who consumes full and so aiding in weight management. Diabetes improves the glycemic profile. As it contains methionine amino acid, it provides energy by improving cell activity. Its antioxidant properties promote gut health and digestion. It's effective in bridging iron deficiency.

#### Soybeans

Soybeans have a glycemic index of 15 and a glycemic load of 4.5, with 446 kcal of protein (36 g), carbs (30 g), and fat (20 g) in 100 g. Soybeans are also high in calcium, iron, phosphorus, and other essential minerals. It is also reasonably priced. Soybean bioactive peptides contribute to the regulation of glucose homeostasis and reduce the risk of Type 2 diabetes and coronary artery disease. It reduces the workload on the kidney or renal function [13].

#### Kidney beans

Red kidney beans, white kidney beans, red speckled kidney beans, and black kidney beans are all available. They are high in phenolics and functional proteins with antioxidant, hypoglycemic, and hypolipidemic properties. A cup of kidney beans has 13.36 g of protein and a glycemic index of 22. Regarding macronutrients, kidney beans include 17% protein, 1% fat, 8% carbohydrate, and an abundance of folate, potassium, and magnesium. The net carbohydrate amount in 100 g of kidney beans is 16.4 g [14].

#### Masoor dhal

With a glycemic index of 25, Masoor dhal helps control glucose in blood and weight management. These are rich in protein (25.8 g), fiber (30.5), and an energy of 345.8 kcal per 100 g of Masoor dhal is present. It also contains iron, folate, thiamine, riboflavin [15].

#### Moong dhal

Moong dhal, split and unsplit, has 6 g of protein. It also contains a lot of vitamins E, C and K. The yellow moong dhal contains 348 kcal, 1.2 g fat, 24.5 g protein, 8.2 g fiber, and 59.9 g carbs. As a result, it promotes weight loss and reduces the risk of cardiovascular disease [16].

### Low glycemic index cereals

#### Barley

The glycemic index of barley is around 25, and it varies for different forms of barley. It contains 354 kcal of calories, 12.5 g of proteins, 73.5 g of carbohydrates, 17.3 g of fiber, and 2.3 g of fat per 100 grams. Barley is enriched with magnesium, potassium, calcium, phosphorous, potassium, and zinc. It is perfect for people with Type 2 diabetes mellitus and can aid with magnesium deficiency and weight loss [17].

#### Broken wheat

100 g of damaged grain has a glycemic index of 41 with 73 g of carbohydrates, 14 g of protein, 2 g of fat, and 12 g of fiber. Because broken wheat has a significant amount of folate, iron, and other essential micronutrients, it is an excellent choice for weight loss and regulating the level of glucose in the blood.

#### Bajra

The glycemic index of bajra is 54. It lowers the chance of developing Type 2 diabetes, cardiovascular disease, and inflammatory illnesses such as rheumatoid arthritis, gout, asthma, IBD, and neurodegenerative diseases. One cup of bajra has 6.11 g of protein, 41.19 g of carbohydrates, 1.74 g of fat, and essential micronutrients such as folate, iron, thiamine, riboflavin, niacin and zinc.

#### Oats

Oats with a glycemic index of 55 aid in lowering blood glucose levels and aids weight loss significantly. Whole grain oat contains many essential nutrients in soluble and insoluble fractions, including proteins, carbohydrates, unsaturated fatty acids, and dietary fiber. Oats' micronutrients include vitamin E, folates, zinc, iron, selenium, copper, manganese, carotenoids, betaine, choline, sulphur-containing amino acids, phytic acid, lignins, lignane, alkyl resorcinols which can enhance the weight loss, regulate blood glucose levels.

### Low glycemic index vegetables

Vegetables like ladies' finger (GI:20), green beans (GI:32), onion (GI:10), cabbage (GI:10), green peas (GI:22), radish (GI:8), brinjal (GI:15), cauliflower (GI:10), tomato (GI: less than 15), carrot (GI:16), cucumber (GI:15) are easily available and is affordable to the majority of the population.

### Low glycemic index fruits

Apple (GI:36), oranges (GI:43), dates (GI:42), pomegranate (GI:18), and guava (GI: appx 12) can be incorporated into the diet, but the quantity must be limited and should be approved by the dietician.

## Role of clinical pharmacist in developing diet charts for individual patients & monitoring schedules

The concept of food pharmacy is a currently emerging sector that is designed to increase the approach and consumption of healthy food options that are more focused on nutritional value. In the past 25 years, the spread of chronic diet-related diseases like obesity, Type 2 diabetes mellitus, hypertension, and other metabolic disorders has increased the mortality rate globally [18,19]. A clinical pharmacist can improve the patient's understanding of nutrition and make the food diet easy to follow, which will render better diet-related health outcomes. It is also essential to provide prevention-focused diet and lifestyle modification to enhance the effectiveness of pharmacological therapy to delay the occurrence of Type 2 diabetes mellitus with cost-effective dietary plans. Previous studies state that there are various barriers for an individual, including economic constraints, geographical access, inadequate knowledge, lack of time, unawareness of cooking methods or equipment used, and lack of social support. A significant decrease in the HbA1c and a notable decrease in HDL-c levels were observed in collaboration with a pharmacist and a dietician in Type 2 diabetes mellitus patients. The patient counseling provided by the clinical pharmacist can extensively improve the degree of dietary self-management [20]. The clinical pharmacist can support the nutritionist by conducting an intensive nutrition assessment considering the patient's age, energy expenditure, the medications the patient takes daily, and clinical status to provide the appropriate micronutrients and macronutrients to avoid over- or nutrition orders.

## Prediabetes & risk conditions promoting the onset of Type 2 diabetes mellitus

Prediabetes or intermediate hyperglycemia is a condition where the blood glucose levels are higher than average but below the threshold of having Type 2 diabetes mellitus. When a person is prediabetic, he may be insulin resistant. Based on age, gender, ethnicity, region of residence, and socio-economic status, the prevalence of prediabetes is varied. 5% to 10% of people with prediabetes develop type 2 DM. Obesity, mainly general and abdominal obesity, is a significant risk factor for prediabetes and its progression to Type 2 diabetes. Other risk factors include a family history of diabetes (first-degree relatives), low HDL-c levels, high systolic blood pressure (SBP), smoking, drinking, and gestational diabetes.

## Information on prevention & management of diabetes mellitus

### The Association of Diabetes Care & Education Specialists (ADCES)

It is a multidiscipline professional organization that advocates diabetic care via novel approaches, management, and assistance. Their website also provides practice and patient tools, research, news, and publications.

### American Diabetes Association

It is an organization that provides research funding, community services, and education on diabetes management and prevention. The association encourages people diagnosed with diabetes mellitus to learn more about the latest advancements for treating DM and various lifestyle options to improve the quality of life with expert healthcare professionals.

### “Do I Have Prediabetes” Prediabetes Awareness Website & Centers for Disease Control – Prevention National Diabetes Prevention Program

These platforms help people perform a safe assessment to know whether they are prediabetic or at risk of developing Type 2 diabetes mellitus and also direct the individual on how to join a well-known and established diabetes management program near them.

## Summary

The common practice among obese or diabetic patients is to regulate the glycemic level by weight loss, i.e., they achieve weight loss by following a certain diet pattern and less energy use, leading to increased appetite and overeating resulting in weight gain. In a low glycemic index diet (LGID), the daily intake of food rich in starch and other forms of sugar like refined carbohydrates, grains, and certain fruits and vegetables is restricted. LGID encourages patients to consume food sources rich in amino and essential fatty acids. Likewise, the patients are asked to take protein and healthy fat sources as their preference. A low glycemic index diet includes eating cruciferous vegetables, rich in indirect antioxidants that can battle cardiometabolic disorders. Existing reports reflect that a low glycemic diet can regulate the HbA1c, FBS, body mass index, and lipid profile in the above population with certain lifestyle modifications. A clinical pharmacist will analyze the medication chart of each patient and will examine the patient's adherence to the medications, glycemic control, and lifestyle modification. Based on the intervention, necessary counseling will be given to patients based on their adherence scale. Identifying the health barriers of each patient will help the diabetes care team to design a personalized care plan.
